# Environmental quality and its impact on total fertility rate: an econometric analysis from a new perspective

**DOI:** 10.1186/s12889-023-17305-z

**Published:** 2023-12-02

**Authors:** Shah Md Atiqul Haq, Mohammad Ashraful Ferdous Chowdhury, Khandaker Jafor Ahmed, Mohammed Thanvir Ahmed Chowdhury

**Affiliations:** 1 Department of Sociology, Shahjalal University of Science and Technology, Sylhet, 3114 Bangladesh; 2 Department of Business Administration, Shahjalal University of Science and Technology, Sylhet, 3114 Bangladesh; 3 Institute for the Study of International Migration, Walsh School of Foreign Service, Georgetown University, 37Th and O Streets, NW, WA DC 20057 USA; 4 Department of Applied Sociology and Social Work, North East University Bangladesh, Sylhet, 3114 Bangladesh

**Keywords:** Ecological footprints, Total fertility rate, HDI, GNI, Econometric analysis, Fixed and random effects, Quantile regression

## Abstract

**Background:**

Environmental quality significantly affects various aspects of human existence. This study employs ecological footprint as a proxy to assess the impact of environmental quality on the TFR, measured as births per woman. This study investigates the extent to which ecological footprint indicators impact on the TFR in across 31 countries between from 1990 to 2017.

**Methods:**

We gathered data on ecological footprints, specifically carbon, agricultural land, grazing land, forest products, and fisheries, from the Global Footprint Network. Information on the TFR, Human Development Index (HDI), and per capita Gross National Income (GNI) were sourced from the World Bank and the United Nations. We applied static panel and quantile regression models to scrutinize the connection between the ecological footprint and TFR, showing how the former influences the latter.

**Results:**

The outcomes reveal that, in both fixed and random effects models, factors including HDI, carbon, and fishing grounds exert a negative influence on TFR, all at a significance level of *p* < 0.01. Conversely, cropland and forest product footprints exhibited a favorable impact on the TFR (*p* < 0.01). Furthermore, GNI per capita positively affected the TFR in both models, with a *p*-value of 0.01. Quantiles regression analysis demonstrated that HDI and carbon footprint had a negative impact on TFR across all quantiles. This statistical significance is maintained for all quantiles, although it is only significant for the carbon footprint up to the 60th quantile, at *p* < 0.01.

**Conclusions:**

This study establishes a negative correlation between specific ecological footprint indicators, such as carbon and fishing grounds, and TFR. Conversely, there was a positive correlation between the footprint of forest products and the TFR. The primary conclusion drawn is that there is heterogeneity in the results regarding the relationship between ecological footprint and TFR. Moreover, the ecological footprint indicators considered in this study did not uniformly influence TFR. Each ecological footprint indicator exhibited distinct effects on the TFR, displaying either positive or negative correlation coefficients. Future research endeavors may delve into how ecological footprints impact other population dynamics, such as mortality and migration.

**Supplementary Information:**

The online version contains supplementary material available at 10.1186/s12889-023-17305-z.

## Introduction

Natural resources, renewable energy utilization, population growth, and the consumption of non-renewable energy sources all exert influences on environmental quality. The deterioration of the environment is notably affected by population growth and the utilization of non-renewable energy sources [1]. Among human activities, population expansion and escalating consumption stand out as pivotal global influencers of the environment, potentially serving as the primary catalysts for ecosystem alterations [2]. These factors also bear significant weight regarding environmental sustainability, which has prompted extensive ecological footprint studies conducted by organizations and scholars. The ecological footprint of any given population, whether at the individual, municipal, or national level, serves as a representation of the environmental repercussions of human resource utilization [3].

The continuous economic and population growth of countries worldwide has led to an increase in their ecological footprint [4]. This ecological footprint can, in turn, give rise to variations in total fertility rates^1^ [5]. Given the gravity of anthropogenic climate change and its associated consequences [6], coupled with the fact that human behavior and practices are deeply ingrained in social contexts, exploring the influence of fertility and consumption patterns on the environment, as Barrett et al. (2020) have undertaken [2], holds significant value. Recent research underscores those societal norms such as childbearing, marriage, and fertility rates wield substantial impacts on biodiversity. For instance, Alola et al. (2019) discovered that higher fertility rates in Canada and the United States exacerbated environmental degradation, whereas the association between marriage and reproduction yielded environmental improvements [5].

In a similar vein, Downey and Hawkins (2008) proposed an exploration of distinctive factors like family size, gender-headed households, and ecological quality to assess their impacts [7]. The results of a study in the United States showed that single-mother households and households with young children tend to emit more harmful pollutants [7]. Meanwhile, research conducted in China revealed an inverse correlation between ecological footprint and population density [8]. However, there remains a gap in our understanding of how ecological footprints change due to factors such as rural-to-urban population shifts, changes in population size, and shifts in age demographics [9]. Surprisingly, only a handful of studies [10, 11] have delved into the micro-level impact of age structures on the natural environment. Adding to this, Qaiser et al. (2021) have pointed out that higher ecological footprint consumption is associated with increased under-five mortality rates, particularly in Asian nations. Their study suggested that a one-unit increase in per capita consumption results in a 7.1526 increase in the probability of child deaths per 1,000 people [12]. The escalating risk to children under five years of age is largely attributed to environmental pollution [13].

The existing body of research has effectively established the interconnectedness of ecological footprints, population growth, and environmental degradation, particularly at macro levels. However, this study identifies a notable research gap by focusing on the specific and relatively understudied relationship between ecological footprints and demographic dynamics, particularly fertility rates. While previous research has underscored the significance of economic, cultural, and demographic factors in explaining ecological footprints [14], there has been a scarcity of dedicated research exploring the direct influence of ecological footprints on demographic elements, specifically fertility rates. This study aims to bridge this gap by investigating how variations in TFR can be attributed to ecological footprint calculations for specific resources, elucidating the direct impact of ecological footprints on fertility rates.

This study contributes to the scientific literature in several key ways. Firstly, it provides empirical evidence on the direct association between ecological footprints and fertility rates, shedding light on a relatively unexplored dimension of the population-environment nexus. By analyzing ecological footprints related to various resources (carbon, cropland, grazing land, fisheries, and forest products) and their impact on TFR, this study offers a nuanced understanding of the intricate relationships between resource utilization and demographic dynamics.

Secondly, the study extends the discourse by considering the influence of HDI and GNI per capita alongside ecological footprints, enriching the analysis with a broader socioeconomic context. This holistic approach allows for a comprehensive evaluation of the factors affecting TFR, moving beyond ecological considerations to encompass economic and social dimensions.

Furthermore, this research carries implications for policy development. The findings provide valuable insights for policymakers by emphasizing the role of ecological footprints in shaping fertility rates. Understanding how resource utilization impacts demographic trends can inform sustainable development strategies and environmental conservation efforts.

The remainder of this paper is organized as follows: Literature review section reviews the pertinent literature; Data sample and methods of analysis section presents details of the data sample and analysis methods; Results section reports descriptive and regression results; and Discussion section discusses the study’s findings. In the final section, we conclude our research by offering policy recommendations, acknowledging the study limitations, and suggesting avenues for future research.

## Literature review

### Ecological footprints

As highlighted by Dasgupta et al. [15], the demand for biosphere products and services dramatically surpasses the environmental capacity to deliver these things sustainably. It has also been suggested that far exceeds the sustainable capacity of the environment to provide them [15]. It has been widely observed that as prosperity and development levels increase, so does the demand for these resources [15–18]. The ecological footprint serves as a measure of a population’s sustainability by quantifying the biologically productive land or natural resources required to support its lifestyle needs. This includes factors such as land for agriculture, fiber production, wood regeneration, carbon dioxide absorption from fossil fuel use, infrastructure for producing goods and services, and waste management [19]. Ecological footprint assessments typically encompass six primary categories of productive land use: grazing, forest, arable land, ocean, carbon footprint, and built-up land.

Ecological footprint is widely recognized as a comprehensive tool for quantitatively monitoring and assessing primary environmental impacts [20, 21]. It provides an accounting system that quantifies society’s demands on the natural environment by enumerating all the natural resources necessary to sustain an economy. A prime example of this resource demand can be observed in the five major growing economies collectively known as the BRICS nations (Brazil, Russia, India, China, and South Africa), where activities such as agriculture, mining, and deforestation significantly contribute to their ecological footprint [22]. The overall ecological footprints of these countries grow due to this consumption to levels showing that they have expanded substantially, signaling an unsustainable reliance on natural resources.

It is important to note that changes in ecological footprint assessments are not uniform or linear; they tend to vary and exhibit asymmetry due to fluctuations in population, per capita GDP in agriculture, resource utilization, and environmental shifts [23, 24]. Both short-term and long-term changes in per capita income, renewable energy use, life expectancy, and population density have been identified as factors influencing ecological footprint estimations [3, 4, 8]. An integrated system for environmental sustainability and ecological footprint calculations has been employed to determine a population’s combined usage of energy, carbon, and water resources [6, 25]. A high ecological footprint reflects significant resource consumption and the resulting adverse environmental impact on society.

### The relationship between ecological footprints and fertility rate

Human behavior plays a crucial role in shaping ecological footprint indicators. Factors such as cultural beliefs, orientations, patterns, consumer responsibility, respect for nature, appreciation of nature’s intrinsic values, and environmental education have all been identified as influential factors in affecting energy consumption and economic growth [19]. These elements arguably impact the sustainability of a given population. For instance, Verhofstadt et al. (2016) [26] explored the link between ecological footprints and subjective well-being to understand sociodemographic factors at play. Their findings suggested that cohabitation and homeownership were associated with higher subjective well-being and reduced ecological impact. They also noted that larger families tended to have a lower ecological footprint per person than smaller ones, highlighting the complex dynamics influencing individuals’ global ecological footprints.

Human actions contribute to environmental degradation through ongoing population growth, increasing settlement density, and inefficient management of land, water, and marine resources [12, 27–29]. Zambrano-Monserrate et al. (2020) emphasized that a high ecological footprint index reflects significant natural resource consumption [3]. In developed economies, the way populations consume resources has been identified as a primary driver of environmental degradation within those populations [23]. They also noted that population growth was no longer the sole driver of environmental deterioration.

Türe [30] conducted a comprehensive study examining the relationships among ecological footprints, the HDI, and fertility rates across 102 countries. His analysis revealed a strong negative association between ecological footprints and HDI, indicating that nations with larger ecological footprints tend to have lower levels of human development. This suggests that higher fertility rates can hinder sustainable development due to the increased resource consumption associated with larger populations. In another European study, Alola et al. [5] presented statistically significant evidence of a positive association between fertility rates and ecological footprints over an extended period. This points to a connection between higher fertility rates and larger ecological footprints, implying a more substantial environmental impact resulting from population growth. The study also highlighted trade policy as a significant factor influencing ecological footprints, revealing a positive link between ecological footprints and trade policy openness. This suggests that countries with more open trade policies tend to have larger environmental footprints. However, it’s important to note that the relationship between fertility rates and ecological footprints can be nuanced, especially in regions characterized by high inequality and poverty [31].

Various factors, such as the overuse of natural resources, reduced capacity to absorb environmental pollutants, loss of biodiversity, increased national production, resource and energy consumption, amplified trade, urbanization, population growth, aging, and population density, exhibit both direct and indirect effects on the calculation of the ecological footprint [32]. Consumption patterns are also significantly shaped by socioeconomic factors. For example, nations with the highest fuel consumption rates, such as the US, China, Russia, Germany, and the UK, are influenced by their increased export/import capacity for fuel. This factor often results in more extensive ecological footprint measurements, especially in terms of carbon intensity [33]. The rate of industrial output also plays a crucial role in consumption patterns and environmental impact. Emerging economies like the BRICS nations tend to engage in substantial exports, which can lead to increased environmental pollution as they often prioritize global market competitiveness over environmental protection [33]. Consequently, the ecological footprints of such nations continue to expand.

Moreover, research indicates that consumer behavior, influenced by socioeconomic characteristics, along with fertility rates, significantly impacts the environment [31]. The relationship between high per capita resource consumption and low fertility rates in certain countries underscores the role of cultural and policy factors in shaping this connection [34].

The relationship between ecological footprint consumption and fertility rates is complex, influenced by variables including economic, cultural, and societal factors. Despite some studies suggesting a negative link [35], others show a positive correlation between fertility rates and ecological footprints [36]. Alola et al. [5] emphasizes the need to increase the use of renewable energy to reduce ecological impact. A greater reliance on renewable energy sources is essential to mitigate environmental effects and promote sustainable development. Conversely, higher consumption of non-renewable energy sources is associated with larger ecological footprints.

In India, significant disparities have been observed in carbon footprints among people residing in different districts and within various economic groups. These disparities not only relate to the size of the carbon footprint but also its composition based on consumption activities [14]. Charfeddine and Mrabet (2017) explored the impact of economic development and social-political factors on the ecological footprints of 15 Middle East and North African countries. They found that sociodemographic variables, such as urbanization, life expectancy at birth, and fertility rates, were associated with lower ecological footprints [37]. Danish et al. (2020) reported that natural resource rents, increased use of renewable energy, and urbanization contributed to reductions in ecological footprints, suggesting a positive impact on environmental quality [38].

The complex relationship between fertility rates and environmental degradation is influenced by multiple factors, including agricultural practices, land tenure systems, and consumer behaviors [37]. High fertility rates can exacerbate resource depletion in areas where a significant portion of the population relies on natural resources for agriculture, animal husbandry, forestry, fishing, and inhabits less productive natural ecosystems in marginal areas [37]. Furthermore, the interplay between fertility and marriage has implications for environmental quality [5]. Downey and Hawkins (2008) argued for evaluating the impact of family size and household headship by males or females on ecological quality [7].

While this literature review primarily focused on studying the effects of socioeconomic and sociodemographic changes on the ecological footprint, the present study takes a different approach. It explores how the ecological footprint, as an environmental indicator, influences fertility, a key demographic measure.

## Theoretical and conceptual framework

Our study acknowledges the crucial significance of the dynamic interplay between population growth, resource utilization, and environmental quality. The impact of population expansion and increased resource consumption on environmental conditions has been widely recognized in the literature [1]. These elements serve as the foundation of our theoretical framework, which aligns with the widely accepted notion that burgeoning populations, combined with elevating resource demands, exert considerable pressures on the environment [2]. This relationship operates at multiple levels, ranging from individual consumption patterns to more extensive national and global tendencies [3].

The ecological footprint is a critical metric within our framework, as it serves as a comprehensive tool for evaluating the environmental impact of human resource utilization [3]. The examination of multiple factors, such as land allocation for agriculture, carbon emissions, and resource requirements, provides a comprehensive overview of the relationship between resource use and demographic patterns [19]. Through a thorough analysis of these components, the ecological footprint offers valuable insights into the complex dynamics of human resource utilization and its impact on the environment.

Considering the aforementioned foundation, our conceptual framework emphasizes the causal relationship between ecological footprints and TFR [5]. The literature underscores this causal link, demonstrating how fluctuations in ecological footprints, resulting from resource consumption and population dynamics, directly impact TFR. These causal connections are further refined by factors such as cultural norms, economic conditions, and policy interventions, all of which are explored in the literature [14].

Considering the comprehensive socioeconomic context, we have given due consideration to the impact of both HDI and GNI per capita. These variables, as discussed in the introduction, play a crucial role in shaping both fertility rates and ecological footprints [14]. It is widely accepted in the literature that socioeconomic conditions, such as income levels and human development, have a profound impact on both fertility decisions and patterns of resource consumption [4].

Furthermore, our research framework investigates the consequences of environmental degradation on biodiversity and ecosystem health. Specifically, we analyze how the ecological footprint contributes to changes in environmental quality, which then affects the distribution of biodiversity. The existing literature highlights the interconnected relationships between ecological footprints, environmental degradation, and biodiversity loss [7]. This underscores the considerable impact that resource consumption exerts on the broader ecological system.

Our research represents a significant contribution to the existing body of knowledge, as it addresses the lack of previous studies on the intricate relationship between ecological footprints and demographic changes, specifically fertility rates. Contrary to previous studies that have explored broader ecological impacts, our research focuses on the direct impact of ecological footprints on fertility rates. This provides new insights into the interplay between population and the environment.

By integrating theoretical and conceptual elements, we have developed a robust analytical framework. This framework, which has been informed by insights from the introduction and literature review, guides data collection, shapes our methodology, and informs the interpretation of results. Importantly, it clarifies the inclusion of independent variables and illuminates the causal pathways that underpin our research, resulting in a structured and comprehensive approach to exploring the complex interrelationships between ecological footprints and population dynamics.

## Data sample and methods of analysis

### Sample countries

This study focused on countries with extensive data available for their ecological footprint indicators and TFR statistics from the years 1990 to 2017. The primary objective was to assess the impact of specific ecological footprint measurements related to critical resources on TFR. To facilitate meaningful comparisons between ecological footprint results and well-established determinants of TFR, the selected countries needed to provide comprehensive data on their HDI values and GNI per capita during this period. Furthermore, the study aimed to create a diverse sample that included developed, developing, and least developed countries from various continents. In the end, 31 countries were selected for this study based on data availability, while those with missing data for any years within the 1990 to 2017 timeframe were excluded.

### Description of dependent and independent variables and data sources

Environmental quality is profoundly impacted by factors such as the depletion of natural resources, increases in carbon emissions, and ecological footprints. This study employs ecological footprints as proxy variables for environmental quality to investigate how changes in environmental quality influence various aspects of human life, including human fertility. For instance, air pollution, an indicator of environmental quality, garners significant attention due to its adverse effects on human health and well-being. Moreover, it plays a role in affecting human fertility, with studies showing that air pollution can lead to reduced fertility rates [39, 40].

In our research, we gathered data from publicly available sources to explore and compare the impacts of socioeconomic dimensions (HDI/GNI) and ecological footprints on the TFR across the 31 countries in our sample. We calculated the mean values of these independent and dependent variables over the study period (as presented in Table 1). Specifically, in this study, we considered TFR as the dependent variable, and the TFR data were sourced from the World Bank (https://data.worldbank.org/). The TFR for a given year represents the average number of children a woman would theoretically have if she were to live through her entire reproductive lifespan, bearing children in accordance with the current age-specific fertility rates. This rate is derived by summing the age-specific fertility rates, typically assessed in five-year intervals.

**Table 1:** Selected countries and mean values of selected dependent and independent variables, 1990–2017

**Country** **(listed alphabetically)**	**TFR**	HDI	**GNI Per Capita (US$)**	**Carbon** **(*gha*)**	**Cropland** **(*gha*)**	**Fishing Grounds** **(*gha*)**	**Forest Products (*gha*)**	**Grazing** **Land** **(*gha*)**
Afghanistan	6.67	.40	1904.44	.093	.269	.00015	.083	.212
Angola	6.48	.494	4290.36	.216	.248	.069	.114	.159
Bangladesh	2.97	.502	2041.79	.163	.278	.0133	.088	.0057
Belgium	1.69	.885	32,912.8	4.508	1.06	.113	.785	.511
Chad	6.94	.352	1145.00	.023	.330	.014	.314	.783
Columbia	2.39	.686	8525.36	.633	.326	.0311	.153	.753
Congo DR	6.60	.391	583.33	.0507	.050	.198	.017	.548
Denmark	1.76	.885	34,102.5	4.16	1.21	.852	1.07	.399
Estonia	1.53	.806	20,523.8	3.70	.657	.095	1.94	.14
Germany	1.37	.889	33,258.9	3.73	.813	.060	.535	.172
Haiti	4.07	.453	2350.36	.119	.280	.011	.110	.041
India	3.071	.527	3033.93	.4085953	.314	.015	.138	.008
Italy	1.320	.846	29,470.3	3.183	.889	.121	.491	.417
Japan	1.396	.868	30,885.0	3.630	.482	.428	.333	.134
Malaysia	2.674	.731	15,442.1	1.863	.652	.362	.553	.127
Mexico	2.64	.720	12,578.2	1.565	.532	.0753	.262	.289
Mozambique	5.647	.336	693.70	.105	.253	.012	.373	.037
Nigeria	6.008	.493	3251.7	.210	.528	.053	.230	.084
Panama	2.716	.746	11,871.0	1.029	.335	.368	.239	.483
Peru	2.860	.692	6751.4	.469	.367	.356	.175	.544
Qatar	2.811	.815	107,932.7	11.182	.642	.187	.136	.411
Rwanda	5.325	.381	993.2	.050	.265	.005	.311	.068
Saudi Arabia	3.808	.771	44,972.1	3.027	.632	.072	.163	.211
Singapore	1.41	.844	54,043.5	5.11	.473	.203	.486	.235
Spain	1.286	.841	25,170.0	2.81	1.03	.383	.366	.184
Sri Lanka	2.274	.710	6119.29	.385	.315	.245	.188	.016
Thailand	1.68	.67	9712.14	1.16	.508	.192	.232	.025
Tunisia	2.344	.665	7006.43	.865	.639	.061	.206	.100
The UK	1.77	.877	30,244.2	3.63	.732	.120	.530	.308
The USA	1.98	.897	41,281.4	6.92	.86	.12	1.10	.354
Venezuela	2.74	.711	12,708.1	1.60	.360	.143	.132	.763
**Total**	3.170	.683	18,682.6	2.14	.52	.161	.380	.275
**Observations**	869	836	818	866	866	866	866	866

Source: Authors’ estimation

As established determinants of TFR, we included HDI and GNI data from the World Bank and the United Nations as independent variables. GNI per capita is reported, converted to US dollars, and divided by the mid-year population, using the World Bank Atlas method for comparing economies. HDI data were obtained from another publicly accessible source (https://hdr.undp.org/en/content/human-development-index-hdi). The United Nations (UN) calculates a country’s HDI value based on three key dimensions: health (life expectancy at birth), education (average years of schooling), and standard of living (GNI per capita).

Data on ecological footprint indicators, the primary independent variables in this study, were sourced from the Footprint Network website (https://data.footprintnetwork.org), a publicly accessible and widely used resource. Our focus was specifically on carbon, cropland, fisheries, forest products, and grazing land as these indicators are commonly employed to gauge the impact of human activity on the environment [19]. According to the Global Footprint Network (2022) definition, the ecological footprint per person of a given country is the total ecological footprint divided by the nation’s total population, measured in global hectares (gha) [41]—the units used in this study. We further broke down these values to provide results for each of the five aforementioned ecological footprint indicators. A higher HDI signifies a better overall quality of life, while a larger gha result for each ecological footprint indicator indicates a more significant environmental footprint. A detailed definition of ecological footprint variables is provided in Additional file 1: Appendix A.

### Regression model specification

#### Static model (Fixed and random effects)

This study applied pooled ordinary least squares (OLS) and static models, such as the fixed effect and random effect, to examine the impact of the ecological footprint indicators on the total fertility rate. These models were used to compare the effects of the ecological indicators on the total fertility rate. The impacts were determined using the following equation:

1 $$\begin{array}{c}\mathrm{Total}\;\mathrm{fertility}\;\mathrm{rate}=f\;(\mathrm{HDI},\;\mathrm{GNI}\;\mathrm{per}\;\mathrm{capita},\mathrm{and}\;\mathrm{the}\;\mathrm{selected}\;\mathrm{ecological}\;\mathrm{footprint}\;\mathrm{indicators})\\\mathrm{TFR}=\mathrm\alpha+{\mathrm\beta}_1\;\mathrm{HDI}+{\mathrm\beta}_2\;\mathrm{GNI}+{\mathrm\beta}_3\;\mathrm{Carbon}+{\mathrm\beta}_4\;\mathrm{Cropland}+{\mathrm\beta}_5\;\mathrm F\mathrm i\mathrm s\mathrm h\mathrm i\mathrm n\mathrm g\;\mathrm G\mathrm r\mathrm o\mathrm u\mathrm n\mathrm d\mathrm s+{\mathrm\beta}_6\;\mathrm{ForestProducts}+{\mathrm\beta}_7\;\mathrm G\mathrm r\mathrm a\mathrm z\mathrm i\mathrm n\mathrm g\;\mathrm L\mathrm a\mathrm n\mathrm d+\varepsilon\end{array}$$

In the above equation, HDI, GNI per capita, and the selected ecological footprint indicators such as Carbon, Cropland, Finishing Grounds, Forest Products, and Grazing Land are included to find the impact of each independent variable on the total fertility rate. This study applied the Hausman test to examine whether the fixed or random effects would be accepted.

#### Quantile regression (QR model)

Because this study considers data for countries with ecological footprint indicators and varying fertility rates, we assumed the OLS results might not be sufficient to make decisions for all the sampled countries. Unlike the OLS, quantile regression (QR) models do not require the error term and the dependent variable to be normally distributed, so they were deemed to be better able to capture the relationships among all the ecological footprint indicators across the distribution of our dependent variable: fertility rate. Quantile regression, a comprehensive method for statistical analysis of linear and non-linear models, is used in various fields like climate [42], agriculture [43], economics [44], and the environment [45], was used to examine whether the effect of ecological footprint indicators on TFR is heterogeneous or homogenous. Quantile regression was preferred over ordinary least squares (OLS) since it is more robust than OLS in the presence of an outlier and does not need the error term to be normally distributed [46]. We applied 20^th^, 40^th^, 60^th^, 80^th^, and 90^th^ quantiles with the following formula:

2 $${y}_{it}={{x}^{\mathrm{^{\prime}}}}_{it }{\beta }_{0}+{\varepsilon }_{it}{Quant}_{\theta }\left({y}_{it}| {x}_{it}\right)={{x}^{\mathrm{^{\prime}}}}_{it }\ {\beta }_{0}$$

Here, *t* denotes time, *i* denotes countries, *y*_*it*_ denotes total fertility rate, *x*_*it*_ denotes the vector of regressors, *β* denotes the vector of parameters to be calculated, *ε* denotes the error term. *Quant*_*θ*_* (y*_*it*_* | x*_*it*_*)* denotes *θ*^*th*^ conditional quantile of *y*_*it*_ given *x*_*it*_. Calculating the *θ*^*th*^ regression quantile, *0* < *θ* < *1*, solves the following problem:

3 $$\genfrac{}{}{0pt}{}{min}{\beta }\frac{1}{n}\left\{ \sum\limits_{{y}_{it}>{\stackrel{\prime}{x}}_{it}\beta }\theta |{y}_{it}-{\stackrel{\prime}{x}}_{it}\beta |+\sum\limits_{{y}_{it}<{\stackrel{\prime}{x}}_{it}\beta }(1-\theta )|{y}_{it}-{\stackrel{\prime}{x}}_{it}\beta |\right\}=\genfrac{}{}{0pt}{}{min}{\beta }\frac{1}{n}\sum_{i=1}^{n}{\rho }_{\theta }{\varepsilon }_{\theta it}$$

Since the quantile regression is more appropriate than the OLS and other static models for testing our hypothesis, this study considered the 20^th^, 40^th^, 60^th^, 80^th^, and 90^th^ quantiles as shown here:

4 $${Q}_{0.20} \left(TFR\right)= {\alpha }_{0.20}+ {\beta }_{\mathrm{0.20,1}}X+ {\beta }_{\mathrm{0.20,2}}HDI+{\beta }_{\mathrm{0.20,3}}\ GNI+ {\varepsilon }_{0.20it}$$

5 $${Q}_{0.40} \left(TFR\right)= {\alpha }_{0.20}+ {\beta }_{\mathrm{0.40,1}}X+ {\beta }_{\mathrm{0.40,2}}HDI+{\beta }_{\mathrm{0.40,3}}\ GNI+ {\varepsilon }_{0.40it}$$

6 $${Q}_{0.60} \left(TFR\right)= {\alpha }_{0.20}+ {\beta }_{\mathrm{0.60,1}}X+ {\beta }_{\mathrm{0.60,2}}HDI+{\beta }_{\mathrm{0.60,3}}\ GNI+ {\varepsilon }_{0.60it}$$

7 $${Q}_{0.80} \left(TFR\right)= {\alpha }_{0.20}+ {\beta }_{\mathrm{0.80,1}}X+ {\beta }_{0.8\mathrm{0,2}}HDI+{\beta }_{\mathrm{0.80,3}}\ GNI+ {\varepsilon }_{0.80it}$$

8 $${Q}_{0.90} \left(TFR\right)= {\alpha }_{0.20}+ {\beta }_{\mathrm{0.90,1}}X+ {\beta }_{\mathrm{0.90,2}}HDI+{\beta }_{\mathrm{0.90,3}}\ GNI+ {\varepsilon }_{0.90it}$$

## Results

### Descriptive statistics and correlation matrix

Table 2 provides a summary of the descriptive statistics for the study variables. Our analysis encompasses a total of 809 observations. When examining the mean scores for ecological indicators, which reflect the quantity of each indicator ‘utilized’ by the population across all 31 countries, the following values were observed: 2.146 for carbon (with a minimum of 0.014833 and a maximum of 15.46453); 0.534 for cropland (ranging from 0.034001 to 1.510438); 0.166 for fishing grounds (spanning from 0.000161 to 1.284220); 0.386 for forest products (with a minimum value of 0.007592 and a maximum of 3.397480); and 0.268 for grazing land (ranging between 0.004017 and 1.018147).

**Table 2:** Descriptive statistics of dependent and independent variables

	**HDI**	**GNI (per capita)**	**Carbon**	**Cropland**	**Fishing grounds**	**Forest products**	**Grazing land**	**TFR**
Mean	0.683703	18,903.47	2.146222	0.534531	0.166911	0.386428	0.268508	3.018769
Median	0.723000	10,960.00	1.243780	0.466526	0.109472	0.247112	0.202630	2.355000
Maximum	0.943000	132,440.0	15.46453	1.510438	1.284220	3.397480	1.018147	8.606000
Minimum	0.192000	270.0000	0.014833	0.034001	0.000161	0.007592	0.004017	1.130000
Std. Dev	0.185598	21,470.20	2.467610	0.288765	0.190537	0.441475	0.231057	1.734063
Skewness	-0.560479	2.122252	2.064965	0.785527	2.368179	3.468344	0.920475	1.077309
Kurtosis	2.212107	9.084272	9.146987	2.975874	10.74675	18.94077	3.067887	2.973133
Jarque–Bera	63.28141	1855.111	1848.625	83.21889	2779.093	10,187.53	114.3961	156.5111
Probability	0.000000	0.000000	0.000000	0.000000	0.000000	0.000000	0.000000	0.000000
Sum	553.1160	15,292,910	1736.293	432.4354	135.0306	312.6200	217.2230	2442.185
Sum Sq. Dev	27.83279	3.72E + 11	4919.991	67.37511	29.33400	157.4795	43.13701	2429.637
**Observations**	809	809	809	809	809	809	809	809

Source: Authors’ estimation

The reported mean TFR value suggests that women in the studied countries, on average, have more than three children. Calculations reveal a range from a minimum of one child to a maximum of eight children. The presence of high skewness and kurtosis values across most variables implies that the data lack symmetry and do not adhere to a bell-shaped distribution. Given the unique nature of this dataset, quantile regression, which does not require the error term to follow a normal distribution, is deemed appropriate.

To explore the relationships between dependent and independent variables, we constructed a correlation matrix, as presented in Table 3. Concerning the dependent variable, TFR, all ecological footprint indicators, except for grazing land, exhibited negative correlations with TFR. Furthermore, the two control variables, HDI and GNI, also displayed negative associations with TFR, with correlation values of -0.866 and -0.475, respectively. Importantly, the low correlation coefficients among all variables suggest a minimal risk of multicollinearity.

**Table 3:** Correlation matrix of selected dependent and independent variables

	**HDI**	**GNI**	**Carbon**	*Cropland*	**Fishing grounds**	**Forest products**	**Grazing land**	**TFR**
HDI	1							
GNI	0.686	1						
Carbon	0.686	0.893	1					
Cropland	0.728	0.509	0.594	1				
Fishing grounds	0.431	0.216	0.252	0.427	1			
Forest products	0.434	0.234	0.407	0.503	0.197	1		
Grazing land	0.161	0.134	0.164	0.094	0.131	0.018	1	
TFR	-0.866	-0.475	-0.519	-0.607	-0.362	-0.363	0.005	1

Source: Authors’ estimation

### Panel model estimations

In this study, we employed static panel models, including pooled Ordinary Least Squares (OLS), fixed effects, and random effects, to investigate the influence of each ecological footprint indicator on TFR. The outcomes of these models are presented in Table 4. While both fixed and random effects models were considered, the results of the Hausman test, which yielded a *p*-value exceeding 0.05, led us to embrace the random effects model. Notably, our findings indicate that certain indicators, such as HDI, carbon, and fishing grounds, exhibited negative and statistically significant impacts on TFR across nearly all models. Conversely, the remaining ecological footprint indicators, namely cropland and forest products, showed positive but less statistically significant effects on TFR. Furthermore, GNI demonstrated a positive and statistically significant influence on TFR in most models, reaching a significance level of 1%.

**Table 4:** Static panel models to examine the effects of independent variables on TFR

Variables	(1)	(2)	(3)
	**Pooled OLS**	**Fixed Effects**	**Random Effects**
	**TFR**	**TFR**	**TFR**
HDI	-0.0153***	-0.0115***	-0.0115***
	(0.000409)	(0.000481)	(0.000458)
GNI	4.07e-05***	2.54e-05***	2.62e-05***
	(3.40e-06)	(2.33e-06)	(2.26e-06)
Carbon	-0.183***	-0.184***	-0.179***
	(0.0295)	(0.0275)	(0.0256)
Cropland	0.525***	0.0454	0.0473
	(0.159)	(0.169)	(0.165)
Fishing grounds	0.121	-0.723***	-0.649***
	(0.170)	(0.222)	(0.213)
Forest products	0.481***	0.171*	0.167*
	(0.0818)	(0.103)	(0.0983)
Grazing land	1.107***	-0.393	-0.223
	(0.126)	(0.249)	(0.230)
Constant	5.893***	6.104***	6.096***
	(0.0728)	(0.162)	(0.205)
Observations	818	818	818
R-squared	0.795	0.729	0.740
Number of idc		32	32

Standard errors in parentheses, *** *p* < 0.01, ** *p* < 0.05, * *p* < 0.1

Source: Authors’ estimation

In our study, we conducted diagnostic tests to address cross-sectional dependence and assess variance inflation factor (VIF), thereby enhancing the robustness of our findings. The results of these tests are presented in Table 5. Specifically, the Breusch-Pagan LM, Pesaran scaled LM, and Pesaran CD cross-sectional dependence tests all clearly indicate the absence of cross-sectional dependence. The probability values associated with these three tests support the acceptance of the null hypothesis, signifying cross-sectional independence. This observation can be attributed, at least in part, to the diverse geographical origins of our dataset. Furthermore, Table 6 reaffirms the absence of multicollinearity, as all our study variables exhibited VIF values below 10.

**Table 5:** Cross-sectional dependence test results

Test	Statistic	Prob
Breusch-Pagan LM	545.1979	0.0625
Pesaran scaled LM	0.546034	0.5850
Pesaran CD	0.781795	0.4343

Source: Authors’ estimation

**Table 6:** Multicollinearity: Variance inflation factor (VIF)

Test	VIF	1/VIF
GNI	6.68	0.149724
Carbon	6.64	0.150638
HDI	3.91	0.256011
Cropland	2.69	0.372092
Forest products	1.63	0.613439
Fishing grounds	1.31	0.764860
Grazing land	1.05	0.951273

Source: Authors’ estimation

### Quantile regression

In this study, we employed quantile regression to explore the varying impact of ecological footprint indicators on TFR across different quantiles. Table 7 provides a comparison of conventional OLS and quantile regression at various quantile levels. The quantile regression analysis revealed a consistent negative influence of carbon on TFR across all quantiles. However, this negative effect remained statistically significant only up to the 60th quantile. This implies that the detrimental effect of a high carbon footprint on TFR is more pronounced in countries with lower and middle TFRs, with less impact in those with higher TFRs.

**Table 7:** Quantile regression models

Variables	(1)	(2)	(3)	(4)	(5)	(6)
	**TFR**	**TFR** **Q20**	**TFR** **Q40**	**TFR** **Q60**	**TFR** **Q80**	**TFR** **Q90**
HDI	-0.0153***	-0.0121***	-0.0150***	-0.0149***	-0.0162***	-0.0191***
	(0.000409)	(0.000433)	(0.000675)	(0.000454)	(0.000680)	(0.000578)
GNI (per capita)	4.07e-05***	2.66e-05***	4.11e-05***	4.44e-05***	4.21e-05***	4.93e-05***
	(3.40e-06)	(3.60e-06)	(5.61e-06)	(3.77e-06)	(5.65e-06)	(4.80e-06)
Carbon	-0.183***	-0.111***	-0.190***	-0.115***	-0.0247	-0.000199
	(0.0295)	(0.0312)	(0.0486)	(0.0327)	(0.0490)	(0.0417)
Cropland	0.525***	0.0366	0.709***	0.0520	0.135	0.444**
	(0.159)	(0.169)	(0.263)	(0.177)	(0.265)	(0.225)
Fishing grounds	0.121	0.793***	0.130	0.0368	-0.227	-0.370
	(0.170)	(0.180)	(0.280)	(0.188)	(0.282)	(0.240)
Forest products	0.481***	0.427***	0.488***	0.369***	0.0983	0.0359
	(0.0818)	(0.0867)	(0.135)	(0.0908)	(0.136)	(0.116)
Grazing land	1.107***	1.104***	0.841***	1.158***	0.848***	0.768***
	(0.126)	(0.133)	(0.207)	(0.139)	(0.209)	(0.178)
Constant	5.893***	4.623***	5.630***	6.091***	6.891***	7.763***
	(0.0728)	(0.0771)	(0.120)	(0.0808)	(0.121)	(0.103)
**Observations**	818	818	818	818	818	818
**R-squared**	0.795					

Standard errors in parentheses, *** *p* < 0.01, ** *p* < 0.05, * *p* < 0.1

Source: Authors’ estimation

Conversely, a high forest product footprint exhibited a positive influence on TFR, particularly noteworthy in countries with lower and middle TFR quantiles. Similarly, grazing land had a positive impact on TFR, with its effects being most modest in the upper quantile of TFR. Notably, the cropland footprint did not significantly affect countries in the middle group, specifically at Q60 and Q80. Its significance was observed only at the second (Q40) and fifth (Q90) quantiles, suggesting that countries with moderate fertility rates are less responsive to cropland dynamics.

Turning to the control variables, HDI and GNI, quantile regression unveiled heterogeneous effects across the distribution of TFR quantiles. A higher HDI consistently exerted a negative impact on TFR, with statistical significance across all quantiles. Moreover, the magnitude of this impact increased as we moved to the upper quantiles. Similarly, a higher GNI had a positive and significant effect on TFR across all quantiles. However, the propensity for an increase was more pronounced in the upper quantiles.

This study evaluated the validity of quantile regression results by conducting a comparison of coefficients among quantile pairs. Table 8 offers insights into this evaluation, presenting the F-test and its associated *p*-value, which play a crucial role in ascertaining the uniformity of coefficients across quantiles. Utilizing a bootstrap approach, we generated a joint distribution that allowed us to effectively compare the estimated coefficients across different quantiles. The outcomes of the F-test clearly reject homogeneity at a significance level of 1% for all quantile pairs. This compellingly demonstrates that the impact of the explanatory variable undergoes notable variation across the entire distribution.

**Table 8:** F test results of quantile regression

Quantile regression results based on carbon		
H_0_ = Q_20_ = Q_40_	H_0_ = Q_40_ = Q_60_	H_0_ = Q_60_ = Q_80_
F(1,810) = 3.94 Probability > F = 0.04	F(1,810) = 2.04 Probability > F = 0.00	F(1, 810) = 5.63 Probability > F = 0.01

Source: Authors’ estimation

**Fig. 1:**
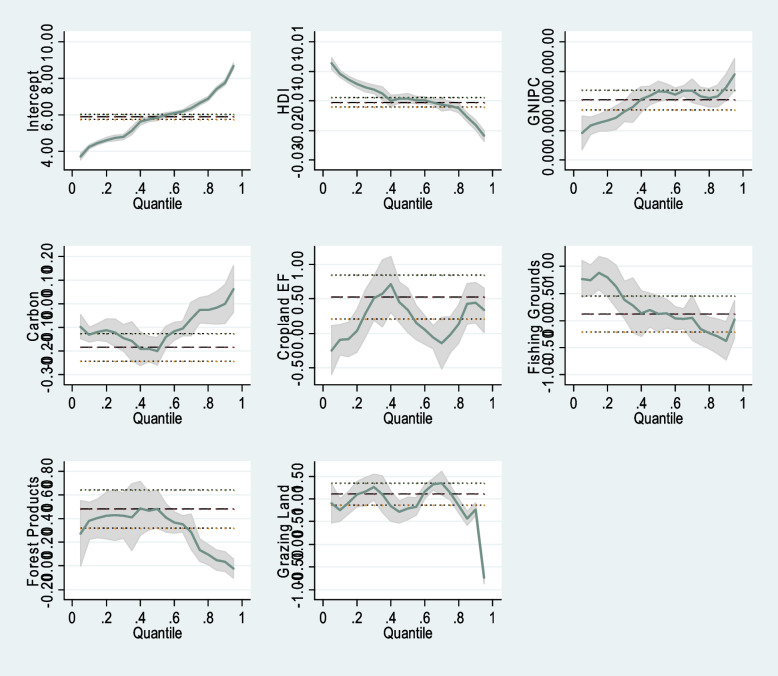
Regression lines: QR vs. OLS for different variables for TFR. Source: Authors’ estimation

To further investigate these relationships, this study aimed to provide graphical representations illustrating the influence of the independent variables on the dependent variable. Figure 1 visually portrays these relationships, clearly demonstrating that most independent variables surpass both the upper and lower significance bounds, as denoted by the dotted lines in each graph. The graphs within this figure vividly depict the heterogeneous nature of the interactions between each indicator and the dependent variable, TFR.

## Discussion

This study delves into the intricate and previously unexplored relationship between ecological footprint indicators and the total fertility rate, drawing from secondary data spanning 31 countries between 1990 and 2017. The selection of countries was purposefully diverse, aiming to ensure ample data availability and maintain a sizable and economically varied sample. The study employs a combination of pooled OLS, fixed effect, and random effect models to scrutinize the influence of HDI, GNI, and crucial ecological footprint metrics—carbon, agricultural land, fishing area, forest products, and grazing area—on total fertility rates. Additionally, to fortify the findings, quantile regression, recognized for its enhanced robustness over OLS, was incorporated.

The results consistently demonstrate a negative correlation between TFR and all ecological footprint indicators, excluding grazing land, even after controlling for HDI and GNI per capita. This negative association reveals that as ecological footprint indicators increase, TFR tends to decrease. This implies that high resource consumption, particularly the depletion of natural resources and heightened carbon emissions, may lead to decreased fertility rates. The analysis also affirms that TFR generally declines with elevated HDI and GNI values, indicating that countries with higher human development indices tend to have lower TFRs. However, for ecological footprint indicators and GNI, the coefficients exhibit negative correlations, albeit with varying degrees.

The static models (fixed and random effects) underscore that both high HDI and substantial carbon footprints exert a negative and statistically significant impact on a country’s TFR (*p* < 0.01). This indicates that fertility rates tend to decrease in countries with higher HDI and larger carbon footprints (gha). This aligns with prior research that identified a long-term connection between fertility and carbon footprint across EU member states [5]. Intriguingly, GNI was positively linked with TFR, signifying that countries with higher GNI, coupled with preferences for higher fertility and gender-specific considerations [37], might exhibit elevated TFRs, particularly in regions with relatively lower HDI values, as noted in the literature review.

Regarding other ecological footprint indicators, both fixed and random effect models highlight a negative influence of fishing grounds’ footprint on TFR (*p* < 0.01). Diminished fish production due to unsustainable fishing practices and environmental harm can precipitate food insecurity, particularly in regions where fish forms a significant component of the diet and a vital protein source. This insecurity, mirrored by a high fishing grounds footprint, may be linked with compromised reproductive health and reduced fertility rates. Conversely, the forest product footprint yields a positive and statistically significant association with TFR. In regions heavily reliant on forest resources, parents may tend to have larger families to help accumulate resources, and strive for more sons to counter any drop in forest resources necessitating alternative income sources [47, 48]. However, the cropland and grazing land footprints failed to register statistically significant impacts in models employing random and fixed effects.

Moreover, constant values with statistical significance (*p* < 0.01) remained consistent across all three models, underscoring the reliability and predictability of these models concerning the influence of each ecological footprint indicator on TFR.

Lastly, employing quantile regression allowed the exploration of the heterogeneous effect of ecological footprint indicators on TFR. Comparative analysis of OLS and quantile regression results illuminated nuanced impacts across different quantiles. For HDI, coefficients with statistical significance at (*p* < 0.01) underscored an inverse effect on TFR across all quantiles (Q20, Q40, Q60, Q80, Q90), with magnified effects in the higher quantiles. This validates prior research indicating that improved health, education, and living standards contribute to reduced TFR in the countries under study. This trend is particularly prominent in higher quantiles, warranting in-depth exploration and modeling of future trends.

Regarding carbon footprints, the study unearthed a negative effect on TFR across all quantiles using quantile regression. However, the negative impact remained statistically significant only up to a quantile of 60. This implies that the detrimental impact of a high carbon footprint on TFR is more pronounced in lower and middle quantiles with lower TFRs than in the upper quantile with the highest TFRs. While carbon footprints generally run high in developed countries [49], individuals may be inclined to limit family size in anticipation of higher CO2 emissions and resultant increased consumption. A high carbon footprint may drive individuals towards smaller families due to concerns about the future and resource scarcity. Conversely, a low carbon footprint may incentivize larger families due to reduced pressure on resources. The results thus reveal a divergence between conventional OLS and quantile regression, particularly for another ecological footprint indicator, cropland. The cropland footprint’s effect on TFR was positive and statistically significant for Q40 and Q90, suggesting contextual variations influenced by socioeconomics and culture. Fishing ground footprints positively affect TFR in lower quantiles, but their impact on TFR is negative, with increasing magnitude, in upper quantiles. Ultimately, the coefficient is positive and statistically significant only for Q20. It has been previously argued that people in countries with low TFR, high HDI, and high GNI (per capita) are aware of the threats associated with the depletion and consumption of their fisheries resources [3]. The people in the countries represented in Q20 may be influenced by the pressure on their fishing grounds, as defined by the high footprint calculation for this resource, to have more children respond to the resulting demands on their livelihood.

Similar to carbon and fishing grounds, the result for the forest products footprint indicator is more pronounced at the lowest TFR quantiles (Q20, Q40, and Q60), and the impact is positive and statistically significant (*p* < 0.01). This positive impact of forest products on TFR for the different quantiles (with variations in the coefficients for the quantiles) could be explained by perceptions of resource scarcity and decisions to have more children for future security. Interestingly, the positive effect of grazing pasture footprint results on TFR is present for all quantiles (*p* < 0.01), with coefficients varying across quantiles. These exceptional results suggest that, regardless of the fertility rate, the reduction in the extent of and the uncontrolled use of pastures for grazing significantly impacts people’s attitudes toward increasing TFR more than the pressures associated with the other ecological footprint indicators. Moreover, the graphical representation in Fig. 1 shows that most independent variables exceed the upper and lower limits of the significance level, indicating heterogeneity in the relationship between ecological footprint and the TFR.

## Conclusion and policy recommendations

Human behavior wields considerable influence over ecological footprint indicators like carbon, crops, fishing grounds, forest products, and grazing land. These ecological footprints intimately tie into environmental quality, which in turn ripples through various facets of human life, including fertility. Numerous studies have pointed to environmental quality indicators such as air pollution as impacting human fertility, with higher levels of environmental pollution notably reducing fertility rates [39, 40]. While prior research, as discussed in preceding sections, often examines the influence of macro-level factors like population dynamics, HDI, per capita income, and economic policies on ecological footprints, this study takes a unique perspective. Here, ecological footprints are considered proxy variables to gauge their impact on another macro variable, the TFR, alongside factors such as HDI and GNI per capita, utilizing an econometric model.

The study’s findings unequivocally reveal that the TFR shares a negative correlation with HDI and GNI per capita, signifying that as HDI and GNI per capita ascend, the TFR tends to decline. Simultaneously, as ecological footprint indicators burgeon, the TFR experiences a dip. Elevated ecological footprints serve as harbingers of resource depletion, heightened consumption, and amplified carbon emissions. Astonishingly, the study also discerns that increased carbon emissions coincide with a lower TFR. Further delving into econometric analysis exposes a rich tapestry of intricacies in the relationship between ecological footprints and TFR. For instance, ecological footprints stemming from fishing grounds appear to usher in a decline in TFR, whereas those rooted in forest products seem to contribute to an upswing in TFR.

In sum, the overarching results of this study paint a compelling picture of a negative correlation between ecological footprint indicators and TFR. In essence, as ecological footprints expand, the TFR tends to dwindle. This expansion flags a heightened exploitation of natural resources. Humans necessitate products and services that, in turn, propel carbon emissions skyward and levy substantial demands on cropland, fishing grounds, grazing land, and forests. Fertility, it seems, responds to this mounting unsustainability encapsulated by the surging ecological footprint measurements, as families contemplate the diminishing natural resource pool and escalating carbon emissions. The prospect of resource scarcity in the future looms large in this context, influencing choices concerning family size. The results garnered from the quantile regression analysis further accentuate the diverse impact of independent variables across the spectrum, showcasing the heterogeneous nature of the relationship between ecological footprint indicators and TFR across the studied countries. Such intricacies merit deeper exploration, potentially through surveys or interviews with prospective or existing parents hailing from countries where the ecological footprint and TFR nexus holds the greatest significance.

Evaluating a population’s ecological footprint serves as a crucial gauge of its capacity to meet human needs and desires in a sustainable manner. Our research has successfully illustrated how the sustainability or lack thereof in resource utilization, encompassing everything from carbon to cropland to fisheries, directly impacts total fertility rates. This insight should be at the forefront of policymakers’ minds, particularly when addressing population dynamics and consumption patterns within a nation’s long-term strategy for sustainable development.

Consider this: if an increase in a population’s fisheries footprint can lead to higher fertility rates within that population, governments have a tangible lever to encourage smaller family sizes by alleviating the strain on their fisheries resources. Recognizing that the perils associated with unsustainable practices wield a quantifiable influence on TFR underscores how sustainability initiatives can be reframed as endeavors to bolster family well-being. Consequently, policies aimed at enhancing family welfare must incorporate ecological footprint assessments as an integral part of the broader context in which tailored programs and projects are conceived and executed.

In essence, this study furnishes a valuable lens through which to comprehend the intricate interplay between ecological footprints and fertility rates. Our findings reveal both positive and negative correlations, shedding light on the multifaceted nature of this relationship, where economic, cultural, and social factors intricately shape the dynamic interaction between ecological footprints and fertility rates, a subject that warrants further in-depth exploration through subsequent studies.

## Limitations

Examining the effect of the ecological footprint on TFR as undertaken in this study represents an initial step in a complex and formidable endeavor. Generalizing findings from this study is challenging due to the multifaceted nature of the impact of ecological footprint indicators, whether positive or negative, on TFR. This impact is contingent not only on variations in HDI and GNI (per capita) but also on the specific resources on which a population relies. The heterogeneity observed in the effect of the ecological footprint on TFR underscores that both the magnitude and direction of this relationship are intricately tied to socioeconomic conditions and cultural contexts.

It is acknowledged that the disparities observed among the 31 countries in this study, particularly in terms of the associations between ecological footprints and total fertility rates, stem from a complex interplay of economic, cultural, social, and demographic factors. This study exclusively incorporates data on HDI and GNI in conjunction with ecological footprint indicators, excluding consideration of other influential socioeconomic, demographic, political, and cultural factors.

This quantitative analysis, while providing valuable insights, is inherently limited in its capacity to comprehensively depict the socioeconomic, cultural, and political dynamics of a given country. Since this study did not consider race, ethnic fragmentation, religion and spatio-temporal context as controls in examining the impact of ecological footprint on total fertility rate and without controlling for them to see their impact on the dependent variable, future research could include these variables if data on these aspects are available. More rigorous econometric models with large data sets could be incorporated in future research to capture the dynamics and robustness of the models. A more thorough understanding of these dynamics would necessitate the collection and in-depth analysis of qualitative data. Future research endeavors are strongly encouraged, particularly those aiming to inform the development of policies that integrate population dynamics with resource preservation, utilizing the ecological footprint as a widely accepted metric of population sustainability.

## Research implications and suggestion for future investigations

This study employs various econometric statistical methods to shed light on the impact of ecological footprints on total fertility rates. These methodologies not only contribute to our understanding of this relationship but also lay the groundwork for future qualitative and quantitative investigations within this domain. Moreover, they can be readily adapted for the study of specific population subsets. Given that countries, as well as smaller population groups, exhibit distinctive sociocultural, socioeconomic, and ecological contexts, along with varying attitudes towards consumption and experiences with nature, in-depth examinations centered on particular nations or groups employing an integrated mixed-methods approach will yield more comprehensive insights into the intricate interplay between ecological footprints and population dynamics. The current study focuses on the impact of ecological footprint on total fertility rate, but future research could use data to analyze how total fertility rate may influence ecological footprints. Since increase or decrease fertility rate can contribute to change in ecological footprints. In addition, this study examines how changes in ecological footprint (carbon, cropland, finishing land, forest products, and rangeland) affect human fertility. This can also be studied using mixed methods. Detailed in-depth interviews can even provide a clear picture of the nexus through an exploratory study. And, a comparative analysis is needed because high fertility countries may provide different results in terms of ecological footprint than low fertility countries.

### Supplementary Information

**Additional file 1: Table. **Detailed definitions of key concepts of Ecological Footprint.

**Additional file 2. **Country data on Ecological Footprints and socio-economic and demographic factors.

## Data Availability

The datasets generated and analyzed during the current study are available on the World Bank and Footprint Network website [https://data.worldbank.org; https://data.footprintnetwork.org].

## Footnotes

1 The sources of the fertility rates employed in this analysis are either registered live births documented by vital registration systems or, where such systems are unavailable, censuses or sample surveys. These rates are generally regarded as dependable indicators of recent fertility patterns. When data on age-specific fertility rates is not available, a model is employed to estimate the proportion of births that occur among adolescents. In the absence of vital registration systems, fertility rates in countries are typically calculated through extrapolation from trends observed in censuses or surveys from previous years.
