# Supplementary material for: Environmental quality and its impact on total fertility rate: an econometric analysis from a new perspective
Source: BMC Public Health. 2023 Dec 2;23:2397. doi: 10.1186/s12889-023-17305-z (PMC10693138; doi:10.1186/s12889-023-17305-z)
Supplement: Supplementary file 1 — Additional file 1: Table. Detailed definitions of key concepts of Ecological Footprint. [file 12889_2023_17305_MOESM1_ESM.doc]

**Appendix A**

**Table.** Detailed definitions of key concepts of Ecological Footprint

| **Indicators** | **Definitions** |
| --- | --- |
| Ecological Footprint | The Ecological Footprint is a metric used to evaluate the area of land and water necessary to produce resources, waste disposal, and maintenance of a particular lifestyle or activity, expressed in global hectares. This assessment encompasses land and sea areas on a global scale due to the international trade of goods and services. In the context of consumption, the term “Ecological Footprint” generally refers to the impact of human consumption on the environment. |
| Cropland Footprint | Cropland is the most productive land-use type to produce bioproducts, which include food, fiber, animal feed, oil crops, and rubber. However, the absence of comprehensive global data sets hinders the ability to accurately calculate the cropland footprint, which fails to consider the negative impact of farming techniques on soil degradation. The cropland footprint encompasses not only crop products allocated to livestock and aquaculture feed mixes, but also fibers and materials. |
| Carbon Footprint | Carbon Footprint is the greenhouse gas emissions from burning fossil fuels and imported goods. It is measured by the land area needed to absorb the carbon dioxide. The Carbon Footprint is the biggest part of the Ecological Footprint, calculated by the forest land needed to offset the emissions. |
| Forest product Footprint | The Forest Product Footprint, which is calculated annually based on the consumption of lumber, pulp, timber products, and fuel wood in a country, is used to assess the impact of forestry on the environment. |
| Grazing land Footprint | The Grazing Land Footprint is determined through an analysis that compares the amount of feed required by all livestock within a specific year to the available supply within a country. The difference in feed demand is then utilized to estimate the contribution of grazing land to the overall feed consumption. |
| Fishing grounds Footprint | The specification of the fishing grounds is deduced by calculating the maximum amount of fish that can be harvested in a sustainable manner for various species. These figures are then converted into an equivalent quantity of primary production, based on the species’ trophic levels, and apportioned among the world’s continental shelves. This estimation encompasses both wild-caught and farmed fish utilized for feed. |
| global hectare (gha) | Global hectares measure the planet’s or a region’s biological productivity for the Ecological Footprint and Biocapacity accounts. This metric, with productivity weighting, reveals the earth’s capacity to sustain life (Biocapacity) and the demand placed on it (the Ecological Footprint). A global hectare is a hectare of land with typical productivity for a given year, which is necessary because of the varying productivity of land types. A global hectare of cropland is smaller than a global hectare of pasture, as more pasture is needed to equal one hectare of cropland. The value of a global hectare may vary yearly due to global productivity changes. |

Source: Global Footprint Network (2022)
